# Paid staff or volunteers – does it make a difference? The impact of staffing on child outcomes for children attending community-based programmes in South Africa and Malawi

**DOI:** 10.1080/16549716.2017.1381462

**Published:** 2017-12-07

**Authors:** Mark Tomlinson, Lorraine Sherr, Ana Macedo, Xanthe Hunt, Sarah Skeen

**Affiliations:** ^a^ Department of Psychology, Stellenbosch University, Stellenbosch, South Africa; ^b^ University College London, UK

**Keywords:** Volunteer, workforce, child well-being, HIV, self esteem, cognitive, stigma, depression, violence, child development, education

## Abstract

**Background:** Globally, and in low and middle income countries (LMIC) specifically, there is a critical shortage of workers. The use of volunteers to support such care delivery systems has been examined, there is scant literature on the impact of volunteers on child outcome in high human immunodeficiency virus (HIV)-affected communities.

**Objectives:** To examine the differential impact of paid versus volunteer workforce in Community Based Organisations (CBOs) providing care to children and families affected by the HIV epidemic in South Africa and Malawi on child outcomes over time.

**Methods:** This study compared child outcomes for 989 consecutive children attending CBOs (0.7% refusal) at baseline and 854 at follow-up (86.3% response rate).

**Results:** Children attending CBOs with paid staff had higher self-esteem, fewer emotional/behavioural problems and less perceived stigma. Likewise, children attending CBOs with paid staff had fewer educational risks, and 20 heightened cognitive performance, and the digit-span memory test. After controlling for outcome at baseline, gender, age, HIV status, and disability, attending a CBO with paid staff remained a significant independent predictor of higher self-esteem scores, less perceived stigma, as well as fewer educational risks and better performance on the drawing test. We found no associations between CBO attendance – paid or volunteer – and children’s depressive and trauma symptoms.

**Conclusions:** Our findings show that in order to most optimally impact on child outcome 30 community-based workers (CBWs) should ideally be paid with trained staff. Specialised input for more severe child difficulties is needed.

## Background

Globally, and in low and middle income countries (LMIC) in particular, there is a critical shortage of workers, notably in community-based care. In order to close the gaps in healthcare access, to address economic hardship, social challenges, conflict, and migration, and in order to promote resilience, a competent workforce is vital. In the arena of health, many LMIC have developed Primary Health Care (PHC) strategies which incorporate the deployment of community health workers (CHWs). CHWs are a broad category of non-professional health personnel who provide the first point of contact between the PHC system, and diverse, often geographically dispersed communities. CHWs are often employed within community-based organisations (CBOs) – all non-government organizations working with HIV affected people in community settings. CBOs also provide a link between communities and the primary health care system. However, the employment of these workers is not limited to the healthcare system, aligned services, including child development and care, also deploy cadres of CHWs. In the broader social and child protection sectors, non-governmental organisations have commonly filled the gap with a workforce often staffed by volunteers in the absence of comprehensive workforce provision, training, funding, and deployment.

Prominent amongst those services delivered by CHSs in South Africa, are those to prevent and treat human immunodeficiency virus (HIV). In a comprehensive paper on the deployment of such workers in the national HIV response, Schneider, Hlophe and van Rensburg [1] noted that, with the growing national support for community-based HIV infrastructure [2], and the inclusion of CHWs in the Comprehensive Care, Management and Treatment Programme governing access to HIV treatment [3], lay health workers have proliferated in the public health sector. The majority of CHWs are employed by government-affiliated non-governmental organisations (NGOs) (where the NGO is granted funds by the government to employ CHWs) [1]. The majority of these workers are oriented towards care rather than disease prevention or health promotion, whereas in other CHW sectors, for instance in early child development, they are more often characterised as prevention workers [1].

There is significant evidence that CHWs contribute to improved population health [4,5]. Countries that use this model include Malawi [6], Ethiopia [7], Zambia [8], South Africa [9], India [10], and Nepal [11]. It has been estimated that there are approximately 60 000 CHWs now working in South Africa [9]. However, there is little consistency in the definition of CHW roles, recruitment, training, and compensation [4]. In South Africa, the national government funds provincial governments who fund NGOs and non-profit organisations (NPOs) to employ CHWs [9,12]. This employment is closer to ‘utilise’ than to ‘employ’, and CHWs work variously as volunteers, part-time staff, for stipends, for non-monetary incentives, or as fully-paid, contracted staff [12].

Volunteers can be broadly categorised into two groups – supplementary and substitute. Supplementary providers encompass all those volunteers who are qualified, and for various reasons and provide their expertise to supplement the work of a chosen agency. There are many examples of such arrangements, including the USA Voluntary Service Overseas Programme (VSO), pro bono legal services [13] . Substitute volunteers differ in the respect that they are often unpaid locals who are brought into an organisation when there is an absence of provision. They may not necessarily be qualified or trained, and their motivations for volunteering may differ. While they may provide necessary services, they may also be seeking experience in order to move on the employment ladder, or may be economically desperate and find the small stipends or meals and transport payments sufficient motivation to volunteer.

There are complexities involved in implementing programmes which rely on community-based workers (CBWs) [5,11]. The major one is that of incentives or payment. There is considerable debate regarding the merits of utilising volunteer as opposed to paid staff. Summarising the arguments in favour of volunteer CHWs, Kaschko [14] notes that key concerns include the fact that, often, funds are not available to pay CHWs; that inconsistent remuneration reduces motivation; that payment may lead to tension between paid and unpaid staff; that altruism is discouraged by payment; and that compensation may lead to unintended consequences for the programme. Exploring these arguments further, it becomes apparent that it is true that in many cases funding bodies and governments do not or cannot, make funding available for salaries for CHWs [7,15]. Traditionally, there are differences in the activities performed by, and duties expected of, paid and volunteer CHWs [16]. It has been suggested that policymakers may be concerned that financial incentives which are perceived as too low, are paid irregularly, or are terminated due to fluctuations in funding may be more of a disincentive to CHWs than no payment at all [17,18]. There is, however, evidence from Haiti and Brazil that irregularities in payment need not be detrimental providing sufficient institutional effort is put into managing available funding and staff motivation [14,19]. Within the HIV response, it is unclear whether the use, or overuse, of volunteers can sustain the response [20,21] and what the longer-term implications are for health systems [22] and treatment roll-out [23].

While a sense of obligation to their communities has been shown to initially mitigate the importance of remuneration to CHWs, once the work has begun, payment often becomes an issue [24]. When they are working on a voluntary basis, and so must maintain other, formal employment in order to earn a living, CHWs have reduced time for administration for their community work duties. For a salaried worker, administrative duties may legitimately take place after hours, but for a volunteer, additional hours will be spent generating income. Salaries not only motivate CHWs, but also encourage greater accountability. Payment has also been shown to have positive repercussions for their esteem and dedication to their work, and capacity for greater geographic reach [6]. Lack of payment, conversely, impacts on staff retention and has a negative impact on staff attendance at training [25,26]. Indeed, a lack of payment has been cited as contributing to the outright collapse of some community health programmes, primarily due to staff attrition [8]. Adequate remuneration increases retention [14] reduce attrition [27]. If they are not paid, then the priority of earning a living elsewhere will inevitably take precedence for CHWs, and this can have negative impacts on programme effectiveness, implementation, and sustainability [28,29]. Paying CHWs has been shown to positively impact their motivation, as well as programme implementation and capacity [30].

From an institutional perspective, the use of volunteers may serve to provide much-needed personnel, and allow for full staffing and wider coverage. Yet, there is a cost in terms of staff development, staff maintenance, and longer-term sustainability [31,32]. Staff commitment may be affected by status as a volunteer compared with a paid employee. Quality control and accountability may also be affected, as would the feasibility of ongoing training and minimum standards. In relation to the response to HIV, CHWs effectively exist on the outskirts of national health systems, and, as an infrastructure, ‘shares many of the managerial challenges (stability, recognition, volunteer vs. worker, relationships with professionals) associated with previous national CHW programmes’ [1,p.179], challenges which may lessen their contribution to an effective response to the epidemic.

Finally, the reliance, and overreliance of CBOs on volunteers may hamper the growth and professionalism of provision. It is unclear to what extent the end user is affected. In spite of growing research on workforce issues related to payment or non-payment, there has been little systematic evaluation of the efficacy of paid versus volunteer CBWs in terms of population outcomes. In this paper we examine the differential impact of paid versus volunteer workforce in CBOs providing care to children and families affected by the HIV epidemic in South Africa and Malawi, on child outcome.

## Methods

This study reports on data from the Child Community Care Study, a longitudinal study on children and families affected by HIV/acquired immune deficiency syndrome (AIDS) enrolled in community-based programmes in South Africa and Malawi. The CBOs were supported by a range of project partners (United Nations Children’s Fund (UNICEF), Save the Children, Bernard van Leer Foundation, Firelight Foundation, World Vision, Comic Relief, Regional Psychosocial Support Initiative (REPSSI), Stop AIDS Now, HelpAge, Diana Memorial Fund, and the AIDS Alliance).

### Participants

The sample included children aged 4–13 years and primary caregivers interviewed at baseline (2011–2012) and followed up 15 months later (2013–2014). Of the 989 participants interviewed at baseline, 854 were followed up (retention rate was 86.3%). Participants were recruited from 28 randomly selected CBOs from comprehensive partner lists of 588 CBOs in South Africa (24 across seven South African provinces) and Malawi (four in the Central Region). Thirteen of the South African CBOs were located in urban areas, and 11 in rural areas. In Malawi, all four CBOs were located in rural areas. At each CBO, we recruited the CBO leader and approximately 35 children and their primary caregivers for interviews.

### Procedure

Child data were gathered at baseline and 15 months follow-up by a combination of self-report, caregiver report, and administration of standardised tests by trained data collectors. Information on CBO structure and services was provided by CBO leaders. Adult participants gave written consent for themselves and their child to participate and children provided assent. Participants were interviewed for approximately 45–60 minutes. Interviews were conducted face-to-face by trained data collectors using mobile phones for data collection [33]. All questionnaires, information, and consent forms were translated and checked with back-translation into Xhosa, Zulu, and Chichewa, and participants were interviewed in the language of their choice. The study received ethical approval from the ethics boards at University College London, United Kingdom (UK) (reference number 1478/002) and Stellenbosch University, South Africa (reference number N10/04/112). The designated head of each CBO was interviewed about the structure, function, and services provided by the CBO. Staff structure, staff pay, and personnel details were gathered using a standardised questionnaire.

### Measures

*Demographic characteristics and health status* included: child’s age, gender, and HIV status based on caregiver report. Child HIV status was coded as positive versus negative or unknown.

*CBO staff (paid or unpaid workers)* was determined by each of the 28 CBO leaders. CBO leaders were asked how many of their staff members were paid, and responses were dichotomised into any (at least one paid worker) versus none (no paid workers, only volunteers).

*Psychosocial outcomes* were measured at baseline and follow-up using standardised scales. Five outcomes were included.

- *Self-esteem* was gathered using the child report Rosenberg Self-Esteem Scale [34], a 10-questions scale with items answered on a 4-point scale – from strongly agree to strongly disagree. The scale ranges from 0 to 30, with summed scores below 15 suggesting low self-esteem.
- *Depressive symptoms* were assessed using the 10-item Children’s Depression Inventory (CDI, [35]) and a summed score ranging from 0 to 20 was generated.
- *Trauma symptomatology* was measured by self-report using the Trauma Symptom Checklist for Children (TSCC) [36]. The scale consists of 10 statements, and symptoms include intrusive thoughts, sensations and memories of painful past events, nightmares, fears, and avoidance of painful feelings. The scales ranges from 0 to 30 and a summed scored was generated.
- *Emotional and/or behavioural problems (or internalised and externalised behavioural problems)* were assessed by caregiver report using a short version of the Strengths and Difficulties Questionnaire (SDQ) [37]. The questions refer to psychological or behavioural difficulties of the child over the last six months on a 3-point scale ranging from not true to very true. A total score was based on the sum of internalising problems (emotional or peer-related problems) and the externalising problems scale (conduct and hyperactivity–attention deficit problems).
- *Perceived stigma* was assessed using a child report measure adapted from the UNICEF report on psychological survey measures for orphans and other vulnerable children [38]. The scale consists of five yes-or-no questions and a summed score was calculated.

Developmental outcomes included educational and cognitive outcomes measured at baseline and follow-up. Educational outcomes were assessed by caregiver report using five questions from the Child Status Index (CSI, 39). The items included school regular attendance, school long-term absence, being in the age-appropriate school grade, school performance, and learning progress. Responses were dichotomised and a summed score of educational risks was calculated. The Ten Question screening tool [40] was used to screen for child developmental disability in the domains of language and speech, cognition, hearing, vision, and motor difficulties. A child screened positive if the caregiver reported impairment on at least one of the 10 questions [40]. Two cognitive measures administered by trained data collectors were employed in the study. Nonverbal cognitive ability was assessed using the draw-a-person test (DAP [41]). This screening test is based on children’s drawings of two human figures, which are coded and marked by a researcher using a scoring classification system. An age-standardised score was recorded for each drawing and mean scores ranging from 40 to 130 were calculated. Attention and working memory were measured by the digit-span task, a subtest of the Wechsler Intelligence Scale for Children (WISC-IV [42],). The task involves recalling a series of dictated digits forwards and other series backwards. An age-standardised and norm-referenced score (based on a reference sample of South African children) was recorded for the two recall conditions [43]. Mean scores ranging from 0 to 20 were computed.

### Data analysis

Analyses were conducted in four steps using IBM SPSS 22.0 on the longitudinal sample (n = 854). First, differences between participants lost to and retained at follow-up were tested using chi-square (for categorical variables) and one-way analysis of variance (ANOVA) tests (for continuous variables). Second, we explored baseline differences between children attending CBOs with and without paid staff on sociodemographic and outcome variables using chi-square and one-way ANOVA tests as appropriate. Third, a series of repeated measures ANOVA analyses was conducted to analyse associations between CBO attendance (with or without paid staff) and change in child outcomes over time. Outcome variables included: self-esteem, depressive symptoms, trauma symptoms, emotional/behavioural difficulties, stigma, educational risks, performance on drawing cognitive test and digit span test. Fourth, in order to further investigate whether CBO attendance with paid staff was associated with more positive psychosocial and cognitive outcomes at follow-up, we ran a series of hierarchical multiple linear regressions for each outcome at follow-up, controlling for the outcome at baseline and relevant cofounders. Model 1 included CBO attendance with paid staff as the independent variable; model 2 also controlled for outcome at baseline, gender, age, HIV status, and disability.

## Results

### Differences between children lost to and retained at follow-up

Follow-up rates were high; of the 989 children that participated at baseline, 854 were followed up (86.3% response rate). Despite this, we explored the differences between children lost to and retained at follow-up. Children lost to follow-up were similar on most variables compared with those retained, including gender, age, HIV status, trauma symptoms, self-esteem, and disability. However, children lost to follow-up were more likely to come from South Africa (*X* ^2^ [1] = 9.59, p = .001), and to have dropped out of school (*X* ^2^ [1] = 7.7, p = .01). Children lost to follow-up also had higher depression scores (F [1, 976] = 4.50, p = .03) while stigma scores at baseline (F [1, 842] = 6.40, p = .01).

### Baseline differences between children attending a CBO with or without paid staff

At baseline, most children were attending CBOs with paid employed staff (73%, n = 722) and 27% of children (n = 267) were attending CBOs run by volunteers only. As shown in Table 1, fewer children from Malawi attended CBOs with paid staff compared with CBOs with volunteers (5.4% versus 43.4%). Children attending CBOs with paid staff were younger than those attending CBOs run by volunteers (mean age = 8.7 years versus mean age = 9.6 years). Additionally, children attending CBOs with paid staff had more emotional or behavioural difficulties (mean SDQ score = 3.3 versus mean SDQ score = 2.16). In contrast, children attending CBOs with any paid employed staff had better performance on the draw-a-person cognitive test (mean score = 88.9 versus mean score = 78.8) and better performance on the digit-span test (mean score = 9.4 versus mean score = 7.2) compared with children attending CBOs with volunteers only. No differences were observed at baseline between children attending CBOs with and without paid staff on gender, HIV status, disability, self-esteem, depressive and trauma symptoms, stigma, or educational risks.

**Table 1.** Sample characteristics according to CBO attendance at baseline.

	Total [n = 989]	Child attends a CBO with paid staff [n = 722]	Child attends a CBO with volunteers [n = 267]	X^2^ or F [df], *p* value
**Country**				
South Africa	834 [84.3%]	683 [94.6%]	151 [56.6%]	213.5 [1], **<0.001**
Malawi	155 [15.7%]	39 [5.4%]	116 [43.4%]	
**Child gender**				
Boy	476 [48.6%]	354 [49.7%]	122 [45.7%]	1.26 [1], 0.28
Girl	503 [51.4%]	358 [50.3%]	145 [54.3%]	
**Child age (years)***M [SD]*	8.91 [2.84]	8.65 [2.89]	9.60 [2.58]	22.1 [1], **<0.001**
**Child HIV status**				
HIV positive	135 [13.7%]	107 [14.8%]	28 [10.5%]	3.11 [1], 0.095
HIV negative or unknown	854 [86.3%]	615 [85.2%]	239 [89.5%]	
**Child disability**				
Any	451 [45.6%]	338 [46.8%]	113 [42.3%]	1.59 [1], 0.22
None	538 [54.4%]	384 [53.2%]	154 [57.7%]	
**Self-esteem***M [SD]*	20.99 [2.87]	20.97 [2.84]	21.04 [2.94]	0.10 [1], 0.75
**Depression***M [SD]*	1.08 [1.65]	1.14 [1.69]	0.94 [1.53]	2.71 [1], 0.1
**Trauma***M [SD]*	3.58 [3.23]	3.60 [3.14]	3.52 [3.45]	0.12 [1], 0.73
**Emotional/behavioural problems***M [SD]*	3.00 [2.38]	3.31 [2.45]	2.16 [1.96]	46.97 [1], **<0.001**
**Stigma***M [SD]*	0.56 [1.06]	0.57 [1.06]	0.56 [1.05]	0.01 [1], 0.92
**Educational risk***M [SD]*	0.78 [1.04]	0.77 [1.04]	0.82 [1.04]	0.53 [1], 0.47
**Drawing test***M [SD]*	86.09 [18.57]	88.92 [17.04]	78.77 [20.28]	54.95 [1], **<0.001**
**Digit span test***M [SD]*	8.77 [3.96]	9.37 [3.93]	7.18 [3.58]	58.61 [1], **<0.001**

CBO: community-based organisations; HIV: human immunodeficiency virus.

Associations between attending a CBO with paid staff compared with volunteers and child outcomes over time are shown in Table 2: the mean scores for most child outcomes at baseline and at 15 months follow-up differed significantly according to whether children attended CBOs with or without paid staff.

**Table 2.** Baseline and 15-month follow-up differences according to CBO attendance [N = 854].

	Self esteem			Depression			Trauma			SDQ problems			Stigma			Educational risks			Drawing test			Digit span		
	Pre	Post	p	Pre	Post	p	Pre	Post	P	Pre	Post	p	Pre	Post	p	Pre	Post	p	Pre	Post	p	Pre	Post	p
Child attends CBO with paid staff [n = 722]	21.07	22.54	.**02**	1.07	0.80	n.s.	3.55	4.07	n.s.	3.30	2.98	.**002**	.51	.43	.**04**	.75	.73	.**001**	89.04	93.02	.**009**	9.39	9.17	**<.001**
Child attends CBO with volunteers [n = 267]	21.09	21.73		.93	.85		3.66	4.14		2.18	2.56		.53	.66		.82	1.05		78.74	87.10		7.06	8.57	

*p* value refers to a series of separate repeated measures; ANOVA analyses showing associations between CBO attendance and each child outcome over time.

CBO: community-based organisations; SDQ: Strengths and Difficulties Questionnaire.

#### Mental health

**Figure 1.**
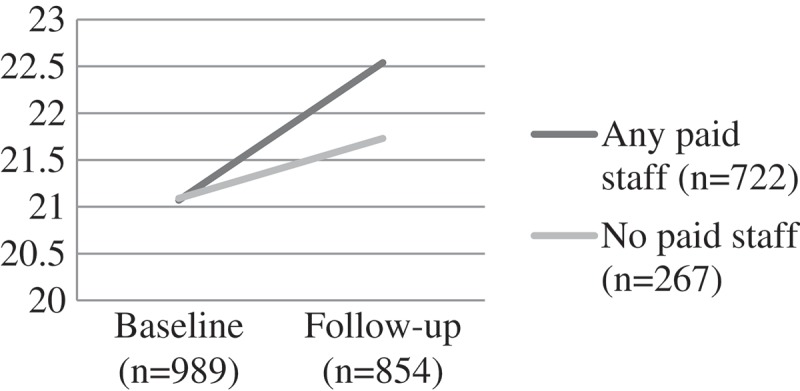
Change over time on child self-esteem scores by CBO attendance, F(1) = 5.52, p = 0.02. CBO: community-based organisations.

**Figure 2.**
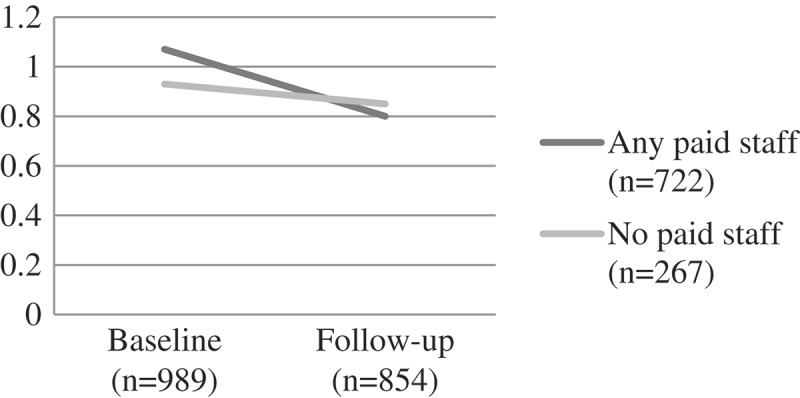
Change over time on child depression scores by CBO attendance, F(1) = 1.17, p = 0.204. CBO: community-based organisations.

**Figure 3.**
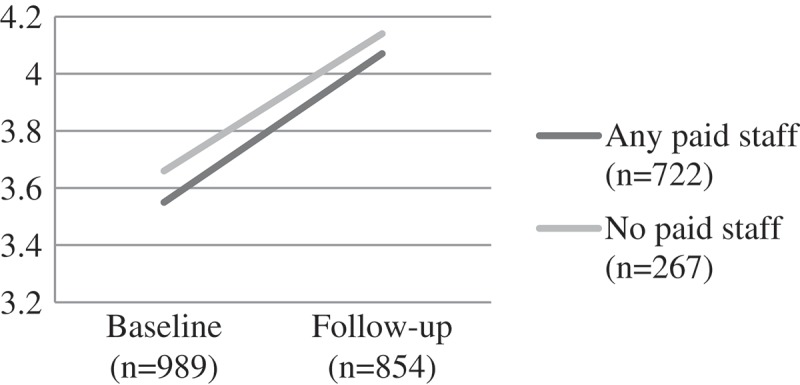
Change over time on child trauma scores by CBO attendance, F(1) = 0.03, p = 0.874. CBO: community-based organisations.

Self-esteem scores for both groups were similar at baseline. Children attending CBOs with any paid staff showed improvements on self-esteem scores over time (from a mean score of 21.07 at baseline to 22.54 at follow-up). Significantly greater improvements were exhibited at follow-up for paid staff, but those cared for by volunteers also improved (F(1) = 5.52, p = 0.02). For the more serious mental health problems there were no significant differences. This was true for child depression (F(1) = 1.17, p = 0.204) where mean scores remained relatively unchanged over time for those attending a CBO with paid workers, and deteriorated over time with those attending volunteer-led CBOs. Similarly, trauma scores got worse over time for both groups – with no significant difference by group F(1) = 0.03, p = 0.874 (Figures 1–3).

#### Emotional and behavioural difficulties

**Figure 4.**
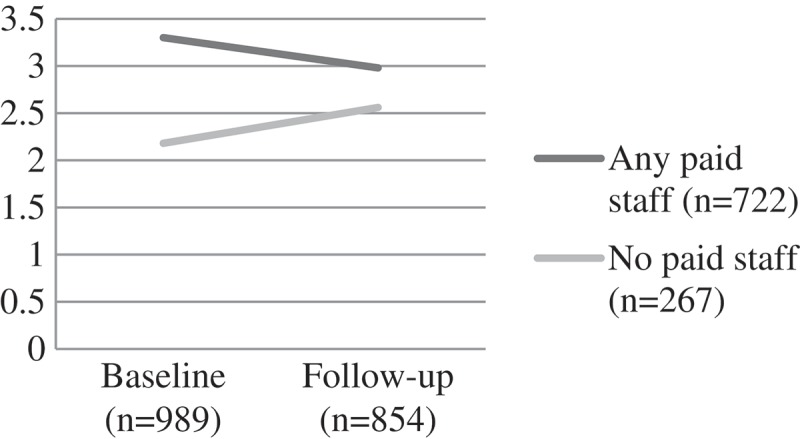
Change over time on child behavioural and emotional scores (SDQ) scores by CBO attendance, F (1) = 9.95, p = 0.002. CBO: community-based organisations; SDQ: Strengths and Difficulties Questionnaire.

Children attending CBOs with any paid staff showed a significant reduction of emotional and behavioural difficulties over time (from a mean score of 3.30 to 2.98 at follow-up). In contrast, children attending CBOs run only by volunteers showed worsening scores over time for emotional and behavioural difficulties (F (1) = 9.95, p = 0.002) (Figure 4).

#### Stigma

**Figure 5.**
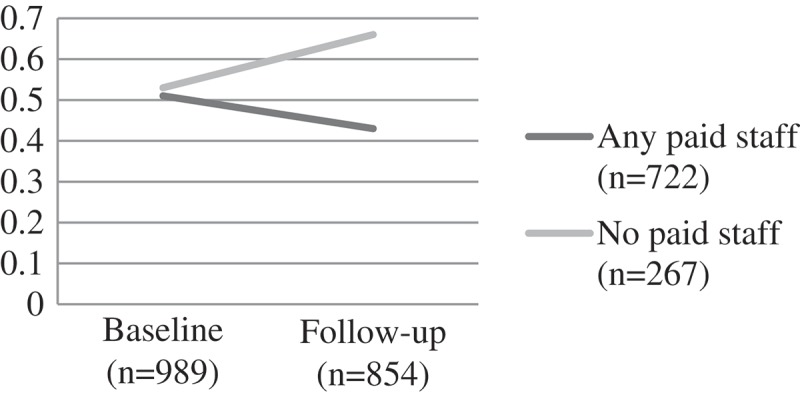
Change over time on child stigma scores by CBO attendance, F(1) = 4.44, p = 0.035. CBO: community-based organisations.

Stigma ratings from the children were similar for both groups at baseline. Over time those attending a CBO with paid staff showed a mean reduction in stigma whilst those with volunteer staff recorded an elevation of stigma over time. The differences by group were significant (F(1) = 4.44, p = 0.035) (Figure 5).

**Figure 6.**
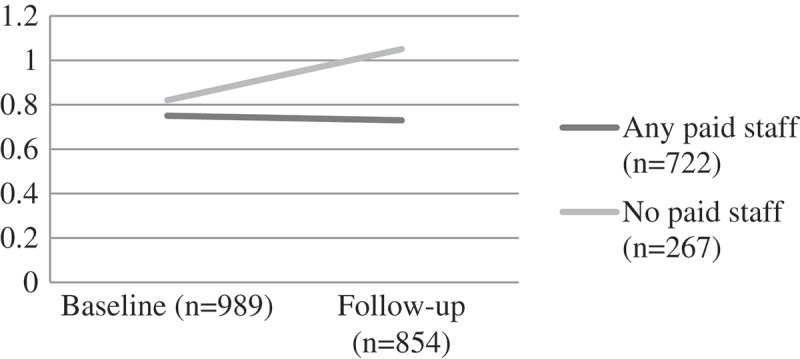
Change over time on child educational risks by CBO attendance, F [1] = 10.78, p = 0.001. CBO: community-based organisations.

**Figure 7.**
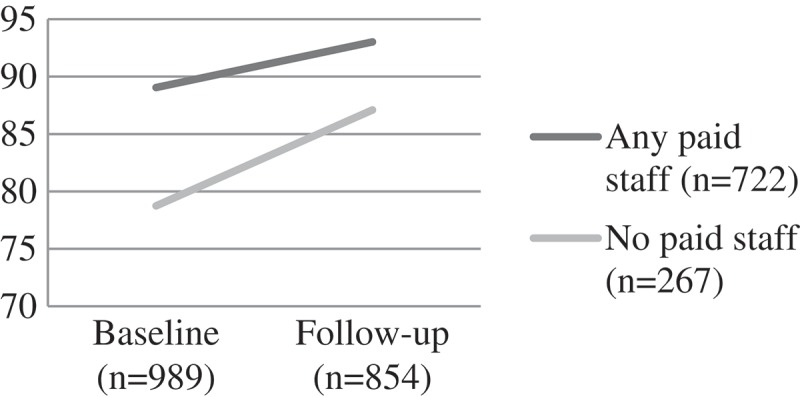
Change over time on child draw-a-man cognitive test score by CBO attendance, F(1) = 6.83, p < 0.001. CBO: community-based organisations.

**Figure 8.**
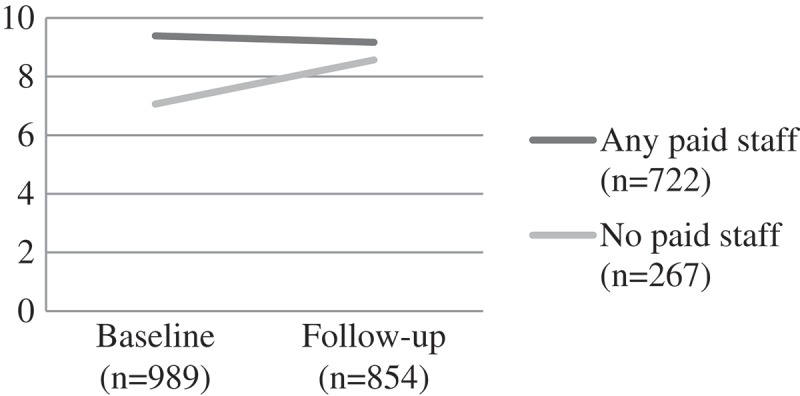
Change over time on digit span score by CBO attendance, F(1) = 30.20, p < 0.001. CBO: community-based organisations.

#### Cognitive performance and educational risks

Educational risks were similar between the groups at baseline. For those attending a CBO with paid workers, the score remained fairly static over time. However, for those attending a CBO with volunteers, educational risks increased over time (F (1) = 10.78, p = 0.001). Children attending CBOs with paid staff had better performance on the draw-a-person cognitive performance test and digit-span test at baseline – suggesting that those with greater need were less likely to be in contact with a CBO with paid workers and more likely to be assisted by volunteers. At follow-up the differences for both digit span (a memory task) (F(1) = 30.20, p < 0.001) and draw-a-person test (an overall cognitive performance task) were significant (F(1) = 6.83, p < 0.001), with performance better at follow-up for the paid worker group (Figures 6–8).

Tables 3 and 4 summarise the hierarchical multiple linear regressions analysing the association between CBO attendance (with or without paid staff) and psychosocial and cognitive outcomes at follow-up controlling for the outcome at baseline and relevant cofounders. Children attending CBOs with paid staff had higher self-esteem (B = 0.83, 95% CI = 0.22, 1.44, p = 0.008), fewer emotional/behavioural problems (B = 0.43, 95% CI = 0.06, 0.80, p = 0.02), and less perceived stigma (B = −0.23, 95% CI = −0.38, = 0.08, p = 0.003) (see Table 3, Model 1). Likewise, children attending CBOs with paid staff had fewer educational risks (B = −0.31, 95% CI = −0.48, = 0.14, p < 0.001), and heightened cognitive performance on both the drawing test (B = 5.89, 95% CI: 3.15, 8.63, p < 0.001), and the digit-span test (B = 0.61, 95% CI = 0.05, 1.17, p = 0.03) (see Table 4, Model 1). After controlling for outcome at baseline, gender, age, HIV status, and disability (Model 2), attending a CBO with paid staff remained a significant independent predictor of higher self-esteem scores (B = 0.78, 95% CI = -.18, 1.40, p = 0.01), less perceived stigma (B = −0.22, 95% CI = −0.37,-0.07, p = 0.04), as well as fewer educational risks (B = −0.25, 95% CI = −0.39, −0.12, p < 0.001) and better performance on the drawing test (B = 3.76, 95% CI = 1.05, 6.47, p = 0.007).

**Table 3.** Longitudinal associations between attending a CBO with paid staff and child psychosocial outcomes.

	Self esteem		Depressive symptoms		Trauma symptoms		Emotional/behavioural problems		Stigma	
	*B [95% CI]*	*p*	*B [95% CI]*	*p*	*B [95% CI]*	*p*	*B [95% CI]*	*p*	*B [95% CI]*	*p*
**Model 1**										
CBO with paid staff	.83 [.22, 1.44]	.**008**	−.05 [-.28, .17]	.66	−.05 [-.61, .50]	.85	.43 [.06, .80]	.**02**	−.23 [-.38, -.08]	.**003**
**Model 2**										
CBO with paid staff	.78 [.18, 1.40]	.**01**	−.07 [-.30, .16]	.57	.13 [-.42, .68]	.63	.04 [-.33, .41]	.83	−.22 [-.37, -.07]	.**004**
Child gender (female)	.01 [-.54, .57]	.97	−.10 [-.31, .10]	.31	.20 [-.28, .68]	.41	−.12, -.44, .21]	.48	.03 [-.11, .17]	.66
Child age (years)	−.06 [-.18, .06]	.35	.01 [-.03, .05]	.53	.09 [.008, .19]	.**03**	−.09 [-.14, -.03]	.**004**	.04 [.006, .07]	.**02**
Child HIV status (HIV+)	−.52 [−1.31, .28]	.21	−.02 [-.32, .28]	.89	1.14 [.43, 1.86]	.**002**	−.04 [-.52, .43]	.86	.02 [-.18, .22]	.85
Child disability	−.39 [-.96, .18]	.18	.06 [-.14, .27]	.54	.29 [-.21, .78]	.25	.26 [-.08, .60]	.13	.15 [.01, .29]	.**01**

B: unstandardised coefficient; CBO: community-based organisations; CI: confidence interval; HIV: human immunodeficiency virus.

Model 1: Univariate regression analyses showing associations between attendance at CBO with paid staff and child psychosocial outcomes; Model 2: Multivariate regression analyses showing associations between attendance at CBO with paid staff and child mental health outcomes, controlling for other predictors: child gender, age, HIV status, disability, and outcome variable at baseline.

*p* < .05, * *p* < .01, ** *p* < .001 ***

**Table 4.** Longitudinal associations between attending CBO with paid staff and child educational and cognitive outcomes.

	Educational risk		Drawing test		Digit-span test	
	*B [95% CI]*	*p*	*B [95% CI]*	*p*	*B [95% CI]*	*p*
**Model 1**						
CBO with paid staff	−.31 [-.48, -.14]	**<.001**	5.89 [3.15, 8.63]	**<.001**	.61 [.05, 1.17]	.**03**
**Model 2**						
CBO with paid staff	−.25 [-.39, -.12]	**<.001**	3.76 [1.05, 6.47]	.**007**	−.29 [-.81, .24]	.28
Child gender (female)	−.23 [-.25, -.10]	**<.001**	.77 [−1.56, 3.10]	.52	.06 [-.39, .51]	.81
Child age (years)	.05 [.03, .07]	**<.001**	-		-	
Child HIV status (HIV+)	−.13 [-.32, .05]	.16	7.37 [3.74, 11.0]	**<.001**	.39 [-.28, 1.06]	.25
Child disability	−.03 [-.16, .11]	.67	.78 [−1.62, 3.17]	.52	−.20 [-.67, .27]	.41

B: unstandardised coefficient; CBO: community-based organisations; CI: confidence interval; HIV: human immunodeficiency virus.

Model 1: Univariate regression analyses showing associations between attendance at CBO with paid staff and child mental health outcomes; Model 2: Multivariate regression analyses showing associations between attendance at CBO with paid staff and child mental health outcomes, controlling for other predictors: child gender, age, HIV status, disability, and outcome variable at baseline.

The cognitive tests (drawing test and digit-span test) are age-adjusted, thus age was not added as a cofounder in the multivariate regression models 2 and 3.

p < .05, * p < .01, ** p < .001 ***.

## Discussion

We found marked differences in child outcome between children attending CBOs with paid staff, and children attending CBOs with volunteer staff. At baseline, children attending CBOs with paid staff had better cognitive outcomes, but worse emotional and behavioural outcomes. Interestingly, those attending CBOs with volunteer staff were significantly younger. It seems that those with greater need may be less likely to have more professional staff while volunteers, who may be less skilled, might be caring for children with more challenges. In addition, despite the sound global evidence on the importance of early child development [38], was the younger children who were more likely to be cared for by volunteer staff.

Over time, children attending CBOs with paid staff showed improvements in self-esteem and a significant reduction of emotional and behavioural difficulties, stigma, and also number of educational risks, while children attending CBOs run only by volunteers showed worsening scores over time for emotional and behavioural difficulties, stigma, and educational risk. Children attending CBOs with volunteers also showed improvements over time on self-esteem and cognitive outcomes, but not at the same rate as children attending CBOs with paid staff. After controlling for a number of factors at follow-up, attending a CBO with paid staff remained a significant independent predictor of higher self-esteem scores less perceived stigma, as well as fewer educational risks and better cognitive performance.

The significance of such changes becomes apparent when one considers the role which perceived stigma, self-esteem, and cognitive capacity play in child well-being. For instance, it is well established that perceived stigma is a significant risk factor for child behavioural difficulties, as well as social isolation [44,45]. For a child social isolation, resulting from perceived stigma, can have deleterious developmental consequences, each cascading from its predecessor (for instance becoming withdrawn in later childhood, and turning to substance abuse in adolescence, which contributes to HIV risk in adulthood). Similarly, educational risks and diminished cognitive performance are risk factors for low educational attainment, poverty, and negative health outcomes [46,47]. For any given child, then, the implications of improvements in these arenas could significantly contribute to their long-term health, and well-being.

This difference in outcomes between the two groups was clear despite the fact that we used a broad definition of payment, and so our group of ‘any paid’ staff is likely diluted by some employees receiving little remuneration. Overall, our findings suggest that the presence of paid staff was a key factor in child outcome in this setting. This is one of the first studies to actively compare outcomes for paid versus non-paid (volunteer) workers in this way.

It is possible that this finding may be due partly to the context in which the intervention took place. Drawing on case study countries, Walt and colleagues [48] examined the feasibility of basing a national primary health care system on volunteers at the community level. They argued that Ministries of Health often assumed that volunteers could be found who would be (a) selected by the community through special meetings or local institutions; (b) supported by the community, in cash or kind, through labour in the fields, surplus produce, drugs sales, or other means; (c) willing to give up a small part of each week to curative or preventive health activities [48]. However, even in Sri Lanka, where contextual factors would favour volunteerism (a relatively autonomous middle class of Buddhist women and long national history of volunteerism), many of these assumptions were in fact not true. In many national programmes workers are neither selected nor supported by community members, and although many are willing to devote time to health activities their usefulness depends on many factors other than willingness [48]. Walt et al. [48] concluded that, although some volunteer programmes have been sustained on a large scale, this can only occur under very specific conditions. These conditions include the presence of large numbers of young, relatively well-educated men and women in rural areas where there are few other opportunities for work, where serving others is a religious or ethical imperative, and where traditional (authoritarian) structures underlie expectations of volunteerism. In contexts such as South Africa, where volunteerism has largely fallen away, conditions in the worst-affected communities may hinder the capacity of young, otherwise eager people, to give of their time without remuneration.

Of note, we found no associations between CBO attendance – paid or volunteer – and children’s depressive and trauma symptoms. These represent the more severe end of child well-being and emotional need. Despite the immense burden of mental disorders in children in LMIC there is a marked lack of services [49,50]. This lack of services includes a lack of specialised personnel to address child mental disorders, as well as a dearth of formal training programmes to enable staff to address child mental health. In answer to this lack of specialised skill, many LMIC have adopted task-sharing approaches to service delivery, including the use of non-specialist workers to deliver mental health services [50,51]. There is growing evidence that lay health workers, if specifically trained, can provide some services and care traditionally delivered by mental health professionals [52–56]. However, it has also been noted that staff working with more severe psychological distress require specialised and ongoing training, and supervision [51]. Our data clearly support such findings, and the need for specialised provision in the youth sector for more severe mental health problems such as depression and trauma needs attention.

Our data suggest that a lack of remuneration hinders the benefit which children can accrue through CBO attendance. By implication, those children who might be at most risk, are least likely to be attending a CBO with paid staff, and in so doing, accessing the most effective care. Furthermore our finding that younger children are more likely to be supported by volunteers flies in the face of the current endorsement of the importance of early child development and the cost effectiveness of early interventions of quality for longer-term future gain.

Our results show that not only does payment appear to be the preferable choice in terms of leading to better outcomes, there is an additional human rights argument for ensuring a paid workforce. In most cases, CHWs in low-income contexts are poor and usually female [57,58]. Expecting economically marginalised women to provide health and welfare services to others, without remuneration, has been argued to amount to labour exploitation [24,59]. Swartz and Colvin [59] suggest that the lack of payment is reflective of a broader, gendered, and structural lack of recognition of the female workforce. The requirement of a fair wage and a secure livelihood is the leading philosophical argument in favour of a paid workforce [7,14]. We provide data that such a philosophy would also have benefits for child recipients of care.

However, while striving for a fully remunerated workforce in all sectors of service provision should be the gold standard towards which LMIC countries strive, the reality is that such services only arose in response to the resource constraint of funding for paid positions. Whether this reflects a genuine dearth, however, or merely a failure to prioritise such informal service providers, is unclear. So, while it is necessary to acknowledge that the prevalence of volunteer service in LMIC may reflect the reality that there was insufficient infrastructure to support the paid service nationwide, it is also necessary to contribute to the development of an evidence base which makes the prioritisation of greater investment in healthcare services an imperative. Until such time as this vision is realised, however, it is equally important to identify strategies to improve the quality of volunteer work. Future work could usefully investigate under which circumstances, and with which populations, volunteer workers are able to make their greatest contribution. However, this should not be in lieu of investment in skilled, paid staff.

### Limitations

The present study is not without limitations, and so our results must be interpreted against the potential pitfalls of non-random allocation of children to groups (either volunteer-staffed CBO, or paid staff CBO). A further potential limitation of this work was our working definition of CBOs with paid staff. CBOs in this category were those with any paid employees. We felt that the existence of any paid employees would add to the professionalisation of the CBO, the skill base, and the quality of service provision. Therefore, the presence of a single paid employee warranted the CBOsʼ inclusion in this category. As such, CBOs who may well have had volunteers on their social workforce may have been included in this group. Thus, our effect size may have been greater had we had a higher threshold by which to consider a CBO as employing paid staff.

## Conclusions

Our findings show that in order to most optimally impact on child outcome CBWs should ideally be paid. We are not suggesting that CBOs staffed by volunteers are not providing essential services or that they are in any way detrimental to the development of children in their care. We do, however, believe that payment is not only a human right imperative but it also appears to be the case that children attending volunteer-staffed CBOs may not have been accessing other higher-level quality services and in some cases appear to have deteriorated over time. Although supportive or supplemental volunteers may have a role, relying entirely on the work of volunteers at CBOs catering to children, needs to be questioned. Our data clearly show the need for developing a community-based workforce in the child and social protection sectors which is not only staffed by paid, skilled individuals, but which also prioritises specialised care for those in need of extra support. This not only allows for skill-building and expertise-gathering when dealing with young children but also provides a career infrastructure and staff development capability that will not only be good for the workforce, but will benefit the recipients as well. We are mindful of the resource constraints that characterise many LMIC particularly in sub-Saharan Africa as well as the political implications of our call for a paid workforce. The political implications of the widespread use of substitution volunteer work must be set against well-intentioned community responsiveness.

## Paper context

Globally, the use of volunteers to support healthcare delivery systems is common, but there is scant literature on the impact of volunteer workers, as opposed to paid staff, on child outcome in HIV affected communities. We compared outcomes for children attending Community Based organisations (CBOs). Our findings show that in order to most optimally impact on child outcome CBWs should ideally be paid with trained staff. Specialised input for more severe child difficulties is needed.
